# Dynamic modeling and simulation of rigid-flexible coupling cable system by absolute nodal coordinate formulation

**DOI:** 10.1038/s41598-022-17731-w

**Published:** 2022-08-09

**Authors:** Xiaoyu Wang, Jingchao Zhao, Haofeng Wang, Huitao Song, Zhong Luo, Qingkai Han

**Affiliations:** 1 School of Mechanical Engineering and Automation, Northeastern University, Shenyang, 110819 People’s Republic of China; 2 AECC Shenyang Engine Research Institute, Shenyang, 110015 People’s Republic of China

**Keywords:** Engineering, Mathematics and computing

## Abstract

The rigid-flexible coupling cable system under large deformation is studied, and the beam element from absolute node coordinate formulation is used to establish flexible cable body of the system. Different numerical integral algorithms are discussed for solving the rigid-flexible cable system and an integration strategy which combines Implicit Euler with Minimum Residual Method (MINRES) is proposed. The influence of the position and number of rigid components and different the lengths of the flexible elements on the system dynamics are analyzed. With constant total mass of the system, higher number of rigid components and their uniform distribution contribute to stabilization of the swing of the flexible cable body. When the total length of the cable is constant, increasing the number of beam elements enhances the nonlinear characteristics of the swing motion and damages the stability. The influence of different factors on the movement of large deformation flexible cable body is obtained through modeling and simulation of the rigid-flexible coupling cable system.

## Introduction

In conventional rigid mechanisms, the transmission of motion and power is mainly realized through joints, which can also be achieved by elastic deformation of the flexible body. The flexible cable can be deemed as a plurality of nodes with large deformation and displacement, withstanding a certain load in the stretching state, which is widely used in various realms such as aerospace and platform hoisting^1–5^. The modeling methods of flexible multi-body system dynamics mainly include floating coordinate method, rotation coordinate method and absolute node coordinate formulation^6–9^. The floating coordinate method, also known as mixed coordinate method, is widely used in the modeling of rigid-flexible coupling system, especially for rigid body small angle movement^10^. The rotating coordination method based on structural kinetics is suitable for small deformation of elastic body and large angle movement of rigid body. The absolute node coordinate formulation (ANCF) is a multi-body systems dynamics analysis method for large deformation and large displacement of flexible body proposed by Professor Shabana in 1996^11^. With respect to large deformation and large rotation of flexible multi-body systems, the infinitesimal rotation angle results in inaccurate rigid body motion model^12,13^. In comparison, ANCF establishes the node position coordinates and slope vector based on the inertial coordinate system, and obtains constant mass matrix without centrifugal acceleration and Coriolis acceleration, which improves the efficiency of numerical calculation^14^ and avoids inertial coupling between the large-scale movement of rigid body and the elastic deformation of flexible body^15^. Shen et al.^16^ established two-dimensional cantilever beam and three-dimensional flexible net based on ANCF, and proposed a method suitable for the dynamic study of large deformation and large displacement flexible cable systems. Chen et al.^17^ derived the rigid-flexible coupling dynamic equations of spatial parallel mechanism under different elastic modulus by Lagrange multiplier method, which provided a method for the rigid-flexible coupling dynamics modeling of spatial mechanism. Liang et al.^18^ studied the modeling of flexible rope and proposed a structure bending spring model, which can simulate ropes with different elastic modulus. Zhao et al.^19^ established the dynamic model of flexible mesh based on the assumption of discrete particle system, and carried out numerical calculation by using the fourth-order Runge Kutta method. Li et al.^20^ derived the dynamic model of compliant mechanisms with nonlinear large deformation components by elliptic integral method and ANCF, and verified the effectiveness of ANCF in the modeling of compliant mechanisms. Zhang et al.^21^ studied the modeling method for the flexible rod with large deformation by ANCF beam element with end deformation constraints. Berzeri et al.^22^ analyzed the planar four-bar mechanism with large deformation components by ANCF.

The dynamics of flexible cable body is studied in this paper considering the influence of rigid components. The rigid-flexible coupling multi-body system is established by ANCF, which contains beam elements and rigid cylinders. The free swing process of the flexible cable under gravity is simulated, during which the kinematics and dynamics of the system are calculated, and the influence of different factors on the swing of the flexible cable body is analyzed.

## Flexible element modeling

**Figure 1:**
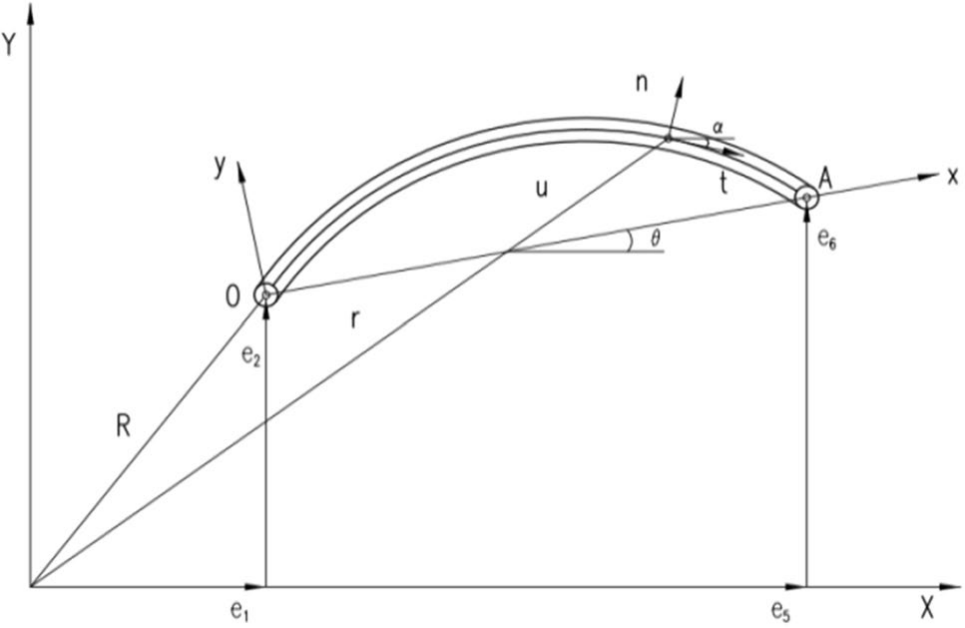
Plane deformation beam element.

In the absolute node coordinate formulation, the node coordinates of the element are defined in the inertial coordinate system. Figure 1 shows a plane deformation beam element based on the ANCF. The nodal coordinates can be approximated by the global shape function. Therefore, the global position of any point on the element can be described by the global shape function and absolute node coordinates expressed as

1 $$\mathbf{r}=\left[\begin{array}{l}{r}_{x}\\ {r}_{y}\end{array}\right]=\mathbf{Se}$$

where, ***S*** is the global shape function. ***e*** is the element node coordinate vector, which can be described by node displacement and slope. ***r*** defines the global position vector of any point.

According to the Euler Bernoulli beam hypothesis, the orientation of the coordinate system is defined by the tangential vector ***t***, and the normal phase vector ***n*** can be described by the transformation matrix under the inertial system.

Where, *r*_*x*_ and *r*_*y*_ are the components of vector **r**. *x* is the coordinate along the undeformed beam axis. The direction of Frenet frame is defined by the angle ***α*** in inertial coordinate system, and the angle can be expressed by the gradient of position vector.

**Figure 2:**
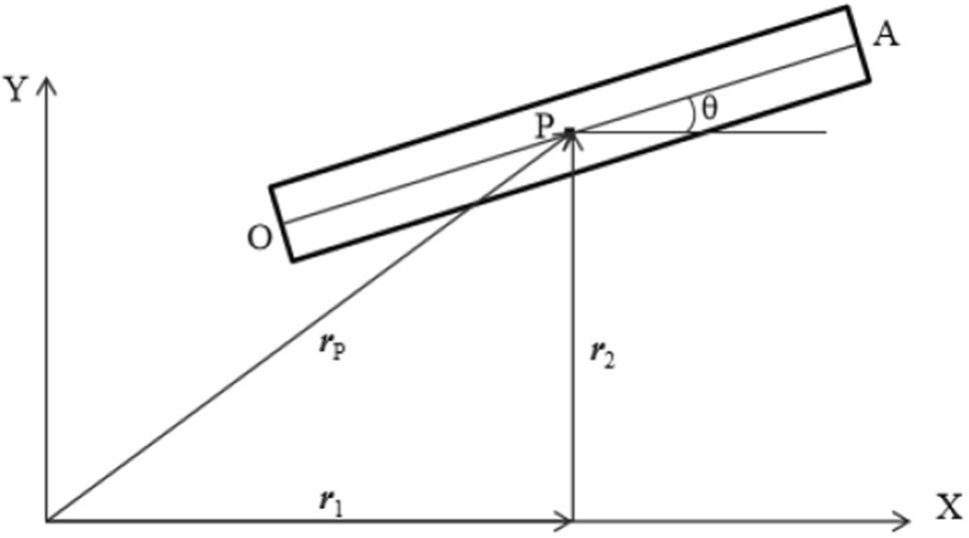
Motion of rigid beam element.

When the beam moves as a rigid body as shown in Fig. 2, the global position vector of any point in the beam element can be expressed as

2 $$\mathbf{r}=\left[\begin{array}{l}{r}_{x}\\ {r}_{y}\end{array}\right]=\left[\begin{array}{l}{R}_{x}+x\;cos\;\theta \\ {R}_{y}+x\;sin\;\theta \end{array}\right]$$

where, *R*_***x***_ and *R*_***y***_ are the global coordinates of end point ***O***. ***θ*** defines the direction of the beam.

The component of vector ***r*** is defined by cubic polynomial in the inertial system. The global shape function containing the complete rigid body mode can be expressed as

3 $$ {\mathbf{S}} = \left[ {\begin{array}{*{20}c} {1 - 3\left( \xi \right)^{2} + 2\left( \xi \right)^{3} } & 0 & {l\left( {\xi - 2\left( \xi \right)^{2} + \left( \xi \right)^{3} } \right)} & 0 & {3\left( \xi \right)^{2} - 2\left( \xi \right)^{3} } & 0 & {l\left( {\left( \xi \right)^{3} - \left( \xi \right)^{2} } \right)} & 0 \\ 0 & {1 - 3\left( \xi \right)^{2} + 2\left( \xi \right)^{3} } & 0 & {l\left( {\xi - 2\left( \xi \right)^{2} + \left( \xi \right)^{3} } \right)} & 0 & {3\left( \xi \right)^{2} - 2\left( \xi \right)^{3} } & 0 & {l\left( {\left( \xi \right)^{3} - \left( \xi \right)^{2} } \right)} \\ \end{array} } \right] $$

The node coordinate vector is expressed as

4 $$ {\mathbf{e}} = \left[ {\begin{array}{*{20}c} {e_{1} } & {e_{2} } & {e_{3} } & {e_{4} } & {e_{5} } & {e_{6} } & {e_{7} } & {e_{8} } \\ \end{array} } \right]^{T} $$

5 $$ \xi = \frac{x}{l} $$

where, *l* represents the length of the element. *e*_1_, *e*_2_, *e*_5_, *e*_6_ represent the absolute node coordinates at ***O*** and A, and the rest node coordinates can be expressed as

6 $$ e_{3} = \frac{{\partial r_{x} \left( {x = 0} \right)}}{\partial x},\;\;e_{4} = \frac{{\partial r_{y} \left( {x = 0} \right)}}{\partial x},\;\;e_{7} = \frac{{\partial r_{x} \left( {x = l} \right)}}{\partial x},\;\;e_{8} = \frac{{\partial r_{y} \left( {x = l} \right)}}{\partial x} $$

According to the kinematics formula given in Eq. (2), the arbitrary rigid body motion is defined by the position of the end point ***O,*** and the element coordinate vector ***e*** can be expressed as

7 $$ {\mathbf{e}} = \left[ {\begin{array}{*{20}c} {r_{ox} } & {r_{oy} } & {\frac{{\partial r_{ox} }}{\partial x}} & {\frac{{\partial r_{oy} }}{\partial x}} & {r_{Ax} } & {r_{Ax} } & {\frac{{\partial r_{Ax} }}{\partial x}} & {\frac{{\partial r_{Ay} }}{\partial x}} \\ \end{array} } \right]^{T} $$

## Dynamic modeling of flexible body

The global displacement of any point of the element can be described by shape function, and the absolute velocity can be obtained by the derivative of displacement with respect to time. Then the kinetic energy of the element can be expressed as

8 $$T=\frac{1}{2}{\int }_{0}^{V}\rho {\dot{\mathbf{r}}}^{T}\dot{\mathbf{r}}dV=\frac{1}{2}{\dot{\mathbf{e}}}^{T}\left({\int }_{0}^{V}\rho {\mathbf{S}}^{T}\mathbf{S}dV\right)\dot{\mathbf{e}}=\frac{1}{2}{\dot{\mathbf{e}}}^{T}{\mathbf{M}}_{a}\dot{\mathbf{e}}$$

9 $$ {\mathbf{M}}_{a} = \int\limits_{V} {\rho {\mathbf{S}}^{T} } {\mathbf{S}}dV $$

where, ***ρ*** and ***V*** are the density and volume of the element, respectively, so the mass matrix is constant. By substituting the shape function, the mass matrix can be expressed as

10 $$ {\mathbf{M}}_{a} = m\left[ {\begin{array}{*{20}c} {\frac{13}{{35}}} & 0 & {\frac{11l}{{210}}} & 0 & \frac{9}{70} & 0 & { - \frac{13l}{{420}}} & 0 \\ 0 & {\frac{13}{{35}}} & 0 & {\frac{11l}{{210}}} & 0 & \frac{9}{70} & 0 & { - \frac{13l}{{420}}} \\ {\frac{11l}{{210}}} & 0 & {\frac{{\left( l \right)^{2} }}{105}} & 0 & {\frac{13l}{{420}}} & 0 & { - \frac{{\left( l \right)^{2} }}{140}} & 0 \\ 0 & {\frac{11l}{{210}}} & 0 & {\frac{{\left( l \right)^{2} }}{105}} & 0 & {\frac{13l}{{420}}} & 0 & { - \frac{{\left( l \right)^{2} }}{140}} \\ \frac{9}{70} & 0 & {\frac{13l}{{420}}} & 0 & {\frac{13}{{35}}} & 0 & { - \frac{11l}{{210}}} & 0 \\ 0 & \frac{9}{70} & 0 & {\frac{13l}{{420}}} & 0 & {\frac{13}{{35}}} & 0 & { - \frac{11l}{{210}}} \\ { - \frac{13l}{{420}}} & 0 & { - \frac{{\left( l \right)^{2} }}{140}} & 0 & { - \frac{11l}{{210}}} & 0 & {\frac{{\left( l \right)^{2} }}{105}} & 0 \\ 0 & { - \frac{13l}{{420}}} & 0 & { - \frac{{\left( l \right)^{2} }}{140}} & 0 & { - \frac{11l}{{210}}} & 0 & {\frac{{\left( l \right)^{2} }}{105}} \\ \end{array} } \right] $$

where, *m* is the mass of the beam element and *l* is its length.

The elastic force of flexible beam element can be obtained by derivation of strain energy with respect to generalized coordinate

11 $$ {\mathbf{Q}}_{k} = \frac{\partial U}{{\partial {\mathbf{e}}}} $$

where, **Q**_***k***_ is the elastic force of flexible beam element, *U* is the total strain energy.

The longitudinal strain caused by longitudinal deformation can be expressed as

12 $$ U_{l} = \frac{1}{2}\int\limits_{0}^{l} {EA\varepsilon_{l}^{2} dx} $$

where,* E* is the elastic modulus of the element. A is the cross-sectional area of the element.

Bending moment *M* can be expressed as

13 $$ M = EI\kappa $$

where, *I* is the second moment of the element cross section. ***κ*** is the curvature of the element.

14 $$ \kappa = \left| {\frac{{d^{2} r}}{{ds^{2} }}} \right| $$

The strain caused by bending deformation can be expressed as

15 $$ U_{t} = \frac{1}{2}\int\limits_{0}^{l} {EI\kappa^{2} dx} $$

The total strain energy of the beam element is

16 $$ U = U_{l} + U_{t} = \frac{1}{2}\int\limits_{0}^{l} {\left( {EA\varepsilon_{l}^{2} + EI\kappa^{2} } \right)dx} $$

The curvature of the beam element can be expressed as

17 $$ \kappa = \left| {\frac{{d^{2} r}}{{ds^{2} }}} \right| = \frac{{\left| {{\mathbf{r}}^{\mathbf{\prime}}} \times {\mathbf{r}}^{\prime\prime} \right|}}{{\left| {{\mathbf{r}}^{\mathbf{\prime}}} \right|^{3} }} = \frac{{{\mathbf{r}}^{\prime \prime T} {\tilde{\mathbf{I}}r^{\prime}}}}{{\left| {{\mathbf{r}}^{\prime T} {{\mathbf{r}}^{\mathbf{\prime}}}} \right|^{\frac{3}{2}} }} = \frac{{{\mathbf{r}}^{\prime \prime T} {\tilde{\mathbf{I}}}r^{\prime}}}{{f^{3} }} $$

where,

18 $$ {\tilde{\mathbf{I}}} = \left[ {\begin{array}{*{20}c} 0 & { - 1} \\ 1 & 0 \\ \end{array} } \right] $$

Longitudinal elastic force **Q**_***l***_ due to longitudinal deformation can be expressed as

19 $$ {\mathbf{Q}}_{l} = \left( {\frac{{\partial U_{l} }}{{\partial {\mathbf{e}}}}} \right)^{T} = \left[ {\int\limits_{0}^{l} {EA\varepsilon_{l} \left( {{\mathbf{S}}^{\prime T} {\mathbf{S^{\prime}}}} \right)dx} } \right]{\mathbf{e}} = {\mathbf{K}}_{l} {\mathbf{e}} $$

20 $$ {\mathbf{K}}_{l} = \int\limits_{0}^{l} {EA\varepsilon_{l} \left( {{\mathbf{S}}^{\prime T} {\mathbf{S^{\prime}}}} \right)dx} $$

where, **K**_***l***_ represents the stiffness matrix corresponding to the longitudinal elastic force. The curved elastic force **Q**_***t***_ due to transverse deformation can be expressed as

21 $$ {\mathbf{Q}}_{t} = \left( {\frac{{\partial U_{t} }}{{\partial {\mathbf{e}}}}} \right)^{T} = \left[ {\int\limits_{0}^{l} {EI\left( {{\mathbf{S}}^{\prime \prime T} {\mathbf{S^{\prime\prime}}}} \right)dx} } \right]{\mathbf{e}} = {\mathbf{K}}_{t} {\mathbf{e}} $$

22 $$ {\mathbf{K}}_{t} = \int\limits_{0}^{l} {EI\left( {{\mathbf{S}}^{\prime \prime T} {\mathbf{S^{\prime\prime}}}} \right)dx} $$

where, **K**_***t***_ represents the stiffness matrix corresponding to the transverse elastic force. The total elastic force **Q**_***k***_ and the total stiffness matrix **K** of the element can be expressed as

23 $$ {\mathbf{Q}}_{k} = {\mathbf{Q}}_{l} + {\mathbf{Q}}_{t} = \left[ {\int\limits_{0}^{l} {EA\varepsilon_{l} \left( {{\mathbf{S}}^{\prime T} {\mathbf{S^{\prime}}}} \right)dx} } \right]{\mathbf{e}} + \left[ {\int\limits_{0}^{l} {EI\left( {{\mathbf{S}}^{\prime \prime T} {\mathbf{S^{\prime\prime}}}} \right)dx} } \right]{\mathbf{e}} $$

24 $$ {\mathbf{K}} = {\mathbf{K}}_{l} + {\mathbf{K}}_{t} = \int\limits_{0}^{l} {EA\varepsilon_{l} \left( {{\mathbf{S}}^{\prime T} {\mathbf{S^{\prime}}}} \right)dx} + \int\limits_{0}^{l} {EI\left( {{\mathbf{S}}^{\prime \prime T} {\mathbf{S^{\prime\prime}}}} \right)dx} $$

where, **S**′ and **S**″ are the first and second derivative of shape function **S** with respect to local coordinates respectively.

25 $$ {\mathbf{S}}\user2{^{\prime}} = \left[ {\begin{array}{*{20}c} {\left[ { - 6\xi + 6\left( \xi \right)^{2} } \right]/l} & 0 & {1 - 4\xi + 3\left( \xi \right)^{2} } & 0 & {\left[ {6\xi - 6\left( \xi \right)^{2} } \right]/l} & 0 & {3\left( \xi \right)^{2} - 2\left( \xi \right)} & 0 \\ 0 & {\left[ { - 6\xi + 6\left( \xi \right)^{2} } \right]/l} & 0 & {1 - 4\xi + 3\left( \xi \right)^{2} } & 0 & {\left[ {6\xi - 6\left( \xi \right)^{2} } \right]/l} & 0 & {3\left( \xi \right)^{2} - 2\left( \xi \right)} \\ \end{array} } \right] $$

26 $$ {\mathbf{S}}\user2{^{\prime\prime}} = \left[ {\begin{array}{*{20}c} {\left[ { - 6 + 12\xi } \right]/l^{2} } & 0 & {\left[ { - 4 + 6\xi } \right]/l} & 0 & {\left[ {6 - 12\xi } \right]/l^{2} } & 0 & {\left[ {6\xi - 2} \right]/l} & 0 \\ 0 & {\left[ { - 6 + 12\xi } \right]/l^{2} } & 0 & {\left[ { - 4 + 6\xi } \right]/l} & 0 & {\left[ {6 - 12\xi } \right]/l^{2} } & 0 & {\left[ {6\xi - 2} \right]/l} \\ \end{array} } \right] $$

The constant mass matrix of flexible elements can be obtained by ANCF without coordinate transformation. The centrifugal force and Coriolis force are zero. In addition, the elastic force is nonlinear in the coordinate system, and the small deformation assumption is generally not used.

According to the first kind of Lagrange equation, the rigid-flexible coupling dynamic equation based on the Lagrange multiplier method is derived as

27 $${\mathbf{M}}_{a}\ddot{\mathbf{e}}+{\mathbf{Q}}_{k}+{{\varvec{\Phi}}}_{\mathbf{e}}^{T}={{\varvec{Q}}}_{a}$$

28 $$\left[\begin{array}{cc}{\mathbf{M}}_{a}& {{\varvec{\Phi}}}_{\mathbf{e}}^{T}\\ {{\varvec{\Phi}}}_{\mathbf{e}}& 0\end{array}\right]\left[\begin{array}{l}\ddot{\mathbf{e}}\\ \lambda \end{array}\right]=\left[\begin{array}{l}{\mathbf{Q}}_{a}-{\mathbf{Q}}_{k}\\ \gamma \end{array}\right]$$

where, **Q**_***k***_ is the elastic force of flexible beam element, which can be obtained by Eq. (11). **Φ**_***e***_ is the Jacobian matrix of the constraint equation. **Q**_***a***_ is the generalized nodal force vector. ***λ*** is Lagrange multiplier. ***γ*** is the right end term of the acceleration equation.

29 $${{\varvec{\Phi}}}_{\mathbf{e}}=\frac{\varvec{\partial}{\varvec{\Phi}}\left(\mathbf{e},{\varvec{t}}\right)}{\varvec{\partial} {\varvec{e}}}$$

30 $${\varvec{\gamma}}=-{{\varvec{\Phi}}}_{tt}-2{{\varvec{\Phi}}}_{\mathbf{e}t}\dot{\mathbf{e}}-{\left({{\varvec{\Phi}}}_{\mathbf{e}}\dot{\mathbf{e}}\right)}_{\mathbf{e}}\dot{\mathbf{e}}$$

## Algorithm analysis

**Figure 3:**
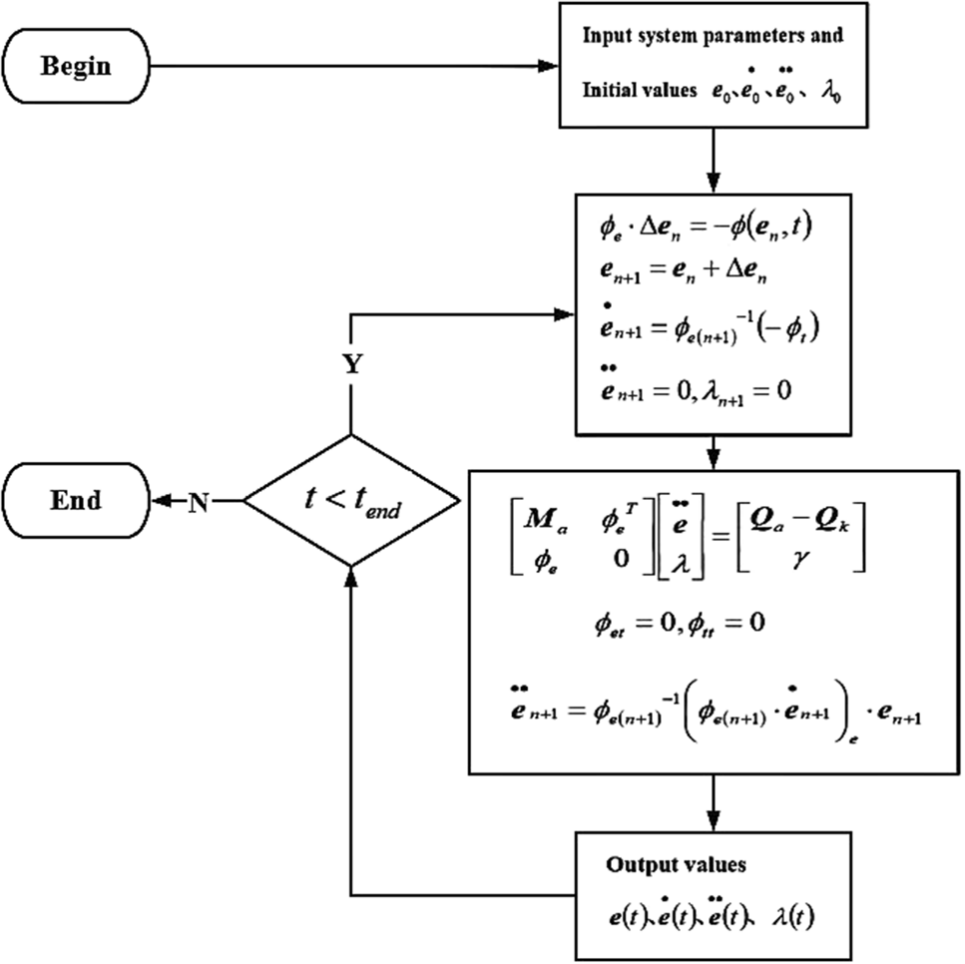
Flow chart of calculation.

Differential algebraic equations (DAEs) of large deformation multi-body systems require numerical integration methods. Some algorithms usually need reasonable selection of parameters to ensure computational convergence and improve computational efficiency. Compared with the explicit algorithm, the implicit algorithm is more stable with higher calculation efficiency. Therefore, the implicit integral method is preferred for the multi-body system dynamics^23^. Runge Kutta algorithm is often used to solve nonlinear ordinary differential equations, however, the Explicit Runge Kutta method has limitations of stabilization regions, so rigid body dynamics equations are generally solved by Implicit Runge Kutta, which requires more computation cost. Compared with the traditional integration methods, Bathe algorithm, as a compound implicit time integration method, is more stable with smaller error accumulation, but the calculation time increases exponentially^24–27^. During the dynamics simulation, the finite element discretization of flexible components produces many pseudo high-frequency responses, which may lead to the non-convergence of Newmark algorithm. And the Newmark algorithm may become unstable when solving long-time nonlinear dynamic problems^28,29^. Besides, the damping factors introduced by Implicit Euler algorithm make the fluctuation amplitude of the curve smaller and the convergence speed faster. Moreover, the mass matrix and stiffness matrix of multi-body system dynamics are large sparse matrices, and MINRES (Minimum Residual Method) is a powerful approximation method for dynamic large sparse matrix^30^. Comparison between GMRES (Generalized Minimal Residual method) and MINRES is to be discussed later. Generally compared with GMRES, MINRES is more stable and can avoid the problem of numerical mutation in GMRES, which is suitable for analyzing the swing movement of the flexible cable. Therefore, in this paper, the rigid-flexible coupling model is calculated by the Implicit Euler algorithm combined with MINRES method. Flow chart of calculation is shown in Fig. 3.

### Algorithm comparison

**Figure 4:**
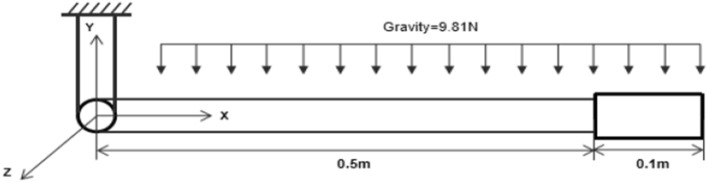
Rigid-flexible coupling model diagram with one rigid body at the end.

In order to verify the solving method of large asymmetric matrices in dynamics equation. MINRES and GMRES are compared with respect to the dynamic system of the cable system with a rigid body attached at the end^16,31^. Parameters are shown in Table 1, and the model diagram is shown in Fig. 4.

**Table 1:** Parameters of the model.

Material	Parameter
Cable length	L1 = 0.5 m
Element diameter	D = 0.01 m
Element length	L = 0.025 m
Cross sectional area	A = 7.85 × 10^−5^ m^2^
Section moment of inertia	I = 4.91 × 10^−10^ m^4^
Elastic modulus	E = 1.0 × 10^7^ Pa
Element density	ρ = 1.0 × 10^3^ kg/m^3^
Rigid-body height	H = 0.1 m

**Figure 5:**
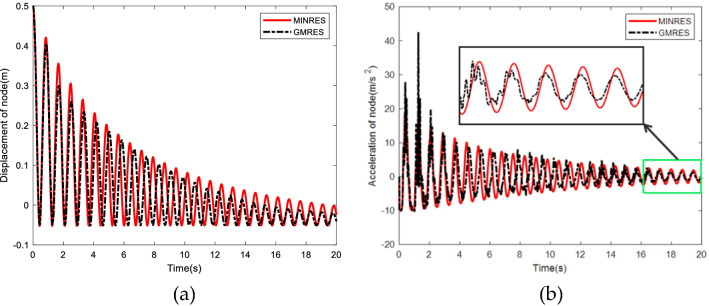
Comparison between GMRES and MINRES. (**a**) Displacement of the beam end node in the Y direction. (**b**) Acceleration of the beam end node in the Y direction.

Total simulation time is 20 s and the time step is 0.005 s. As can be seen from Fig. 5a, the difference of the end node displacement is similar and the convergence speed of GMRES method is slightly faster. Figure 5b shows that with the increase of simulation time, the acceleration tends to be consistent. The curve of MINRES method is smoother, while GMRES method shows abrupt changes at the first several periods and exhibits high frequency fluctuations compared with MINRES. Therefore, when analyzing the swing movement of the flexible cable, MINRES method is more stable.

**Figure 6:**
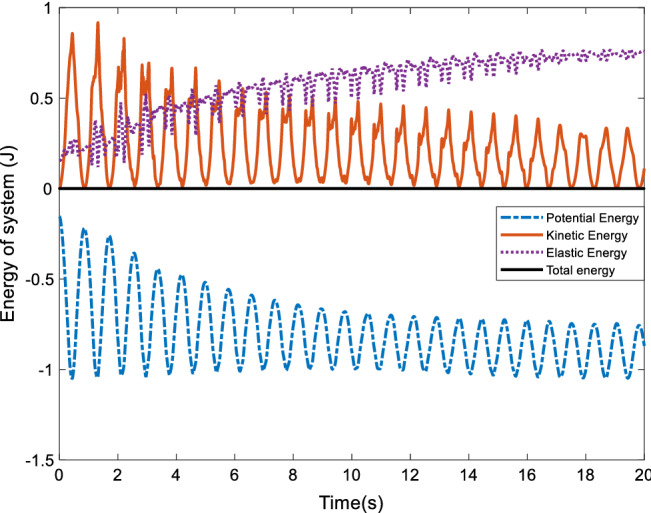
System energy analysis.

The rigid-flexible coupled sing system is only driven by gravity, so the total energy of the system is conserved, which includes the kinetic energy and potential energy of the flexible components and the rigid bodies as well as the elastic deformation energy of the flexible components. Taking the one rigid body model as an example, the energy of the system is analyzed. The change of energy components of the system is shown in the Fig. 6, which implies that the energy is always conserved during the whole simulation process.

### Model validation

**Figure 7:**
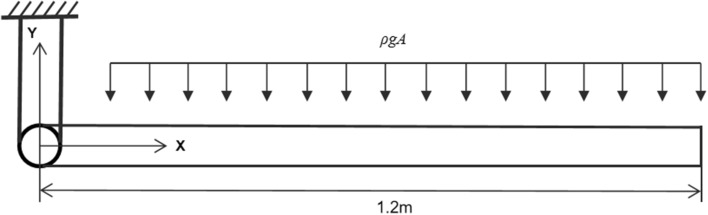
Free falling flexible pendulum.

**Figure 8:**
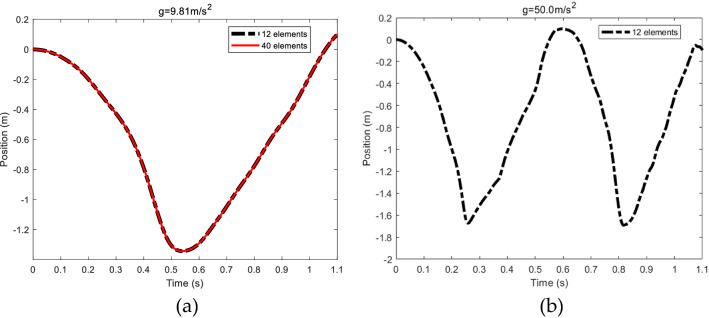
Vertical position of the free end of the pendulum. (**a**) The beam using 12 elements and 40 elements, g = 9.81 m/s^2^. (**b**) The beam using 12 elements, g = 50.0 m/s^2^.

In this section, an example is considered in order to validate the flexible beam model, which is a two-dimensional free falling flexible pendulum under its own weight^32^, as shown in Fig. 7. The beam is connected to the ground by a pin joint at one end. The length, cross section area, second moment of area, density and modulus of elasticity of the beam are 1.2 m, 0.0018 m^2^, 1.215 × 10^−8^ m^4^, 5540 kg/m^3^, 0.7 × 10^6^ Pa, respectively. In the original configuration, the beam is horizontal without velocity. Two cases are considered in the analysis of the falling pendulum. In the first case, the beam is assumed to fall under normal gravity force, while in the second case gravitational acceleration is increased to 50.0 m/s^2^. Figure 8a shows the position of the tip point of the beam using 12 and 40 finite elements for the first case. It is clear that there is a good agreement between the two models, which demonstrates that the solution converges with small numbers of elements. Figure 8b shows the large deformation result of the falling beam under gravitational acceleration of 50.0 m/s^2^ using 12 finite elements. The results presented in Fig. 8 agree with those in the literature^32^.

**Figure 9:**
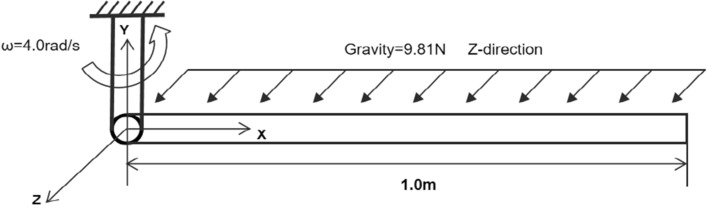
Flexible pendulum model in three dimensional motion.

In addition to the above two-dimensional beam example, the flexible pendulum in the case of three-dimensional motion is considered in this second example^33^. The flexible pendulum is under gravity with initial angular velocity about the vertical Y-axis. Figure 9 shows the model setup, and the model simulation parameters are shown in Table 2.

**Table 2:** Parameters of flexible pendulum.

Property	Value
Cable length	L = 1.0 m
Element density	ρ = 3.20 × 10^2^ kg/m^3^
Elastic modulus	E = 1.60 × 10^6^ N/m^2^
Cross sectional area	A = 2.50 × 10^−5^ m^2^
Poisson’s ratio	0
Initial angular velocity	ω = 4.0 rad/s
Step size	0.0001 s
Simulation time	2.0 s

**Figure 10:**
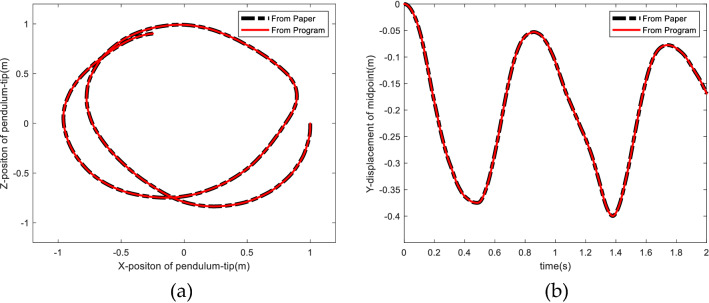
Comparison between simulation results and the results of former literature. (**a**) The Z versus X position of the tip of the pendulum. (**b**) The Y position of the mid-point of the pendulum.

Figure 10 shows the comparison between our ANCF cable method simulation results and the results in the literature, it can be seen, the two results are consistent, which verifies the ANCF cable element method in this paper.

## Results and discussion

### Influence of number of rigid bodies

Rigid bodies are added to the flexible cable to establish a rigid-flexible coupling model. Assuming the total weight of the model and parameters of flexible cable remains the same, only the number and distribution of rigid bodies are changed, therefore, two rigid-body, three rigid-body and five rigid-body models are constructed. The process of the falling and swinging of the rigid-flexible cable system freely from the horizontal state is simulated and the movement of the rigid body connected at the end of the flexible cable is analyzed. Parameters of rigid cylinder are shown in Table 3.

**Table 3:** Parameters of rigid cylinder.

Rigid-body	Parameter
Cylinder_1 height	H1 = 0.1 m
Cylinder_2 height	H2 = 0.05 m
Cylinder_3 height	H3 = 0.025 m
Cylinder diameter	D = 0.04 m
Cylinder density	ρ = 1.0 × 10^3^ kg/m^3^
Section moment of inertia	I = 1.26 × 10^−7^ m^4^

**Figure 11:**
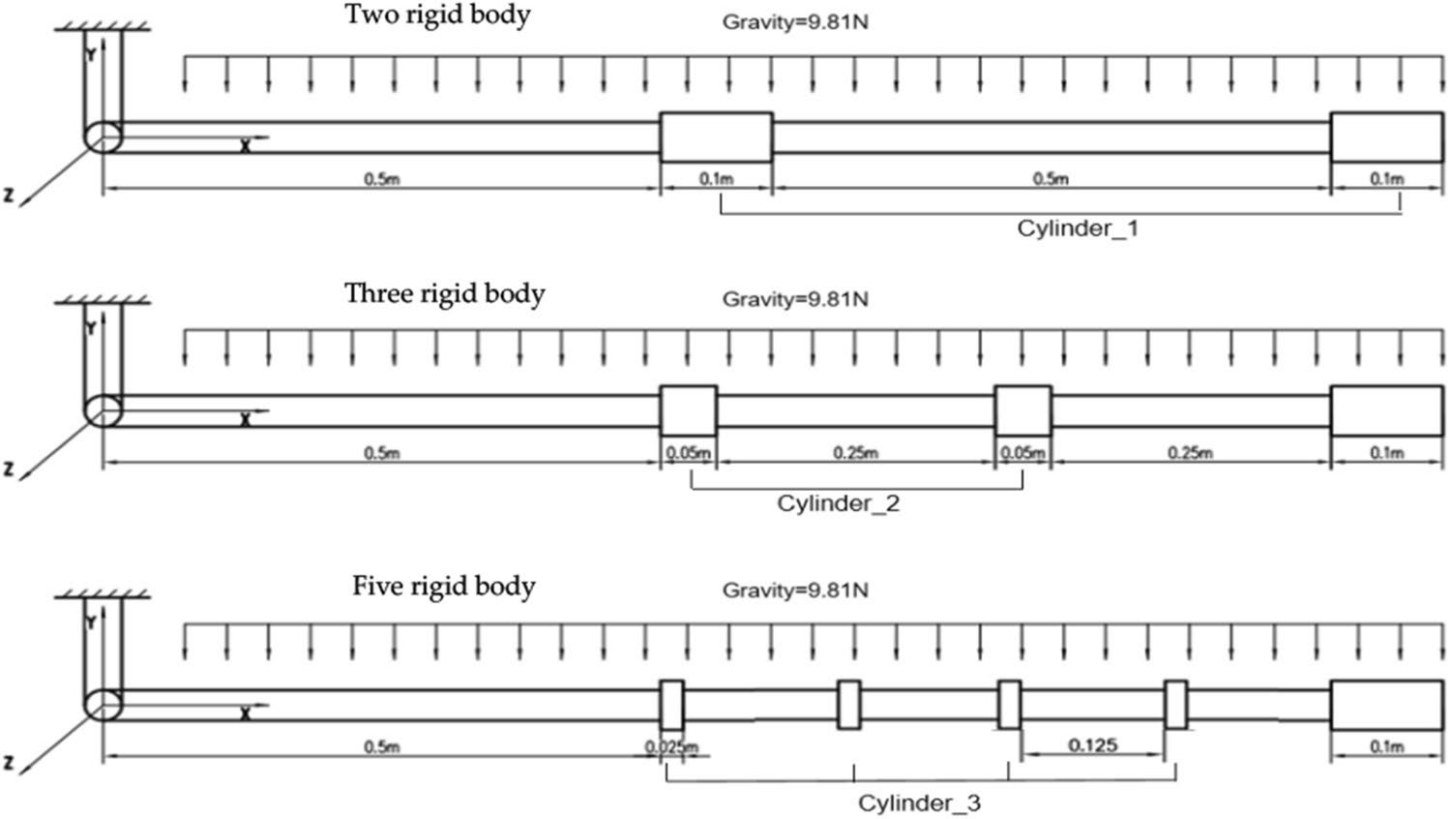
Schematic diagram of different patterns of distribution of rigid bodies.

Figure 11 shows three patterns of rigid-flexible coupling models with different numbers of rigid bodies, including two rigid-body model, three rigid-body model and five rigid-body model.

**Figure 12:**
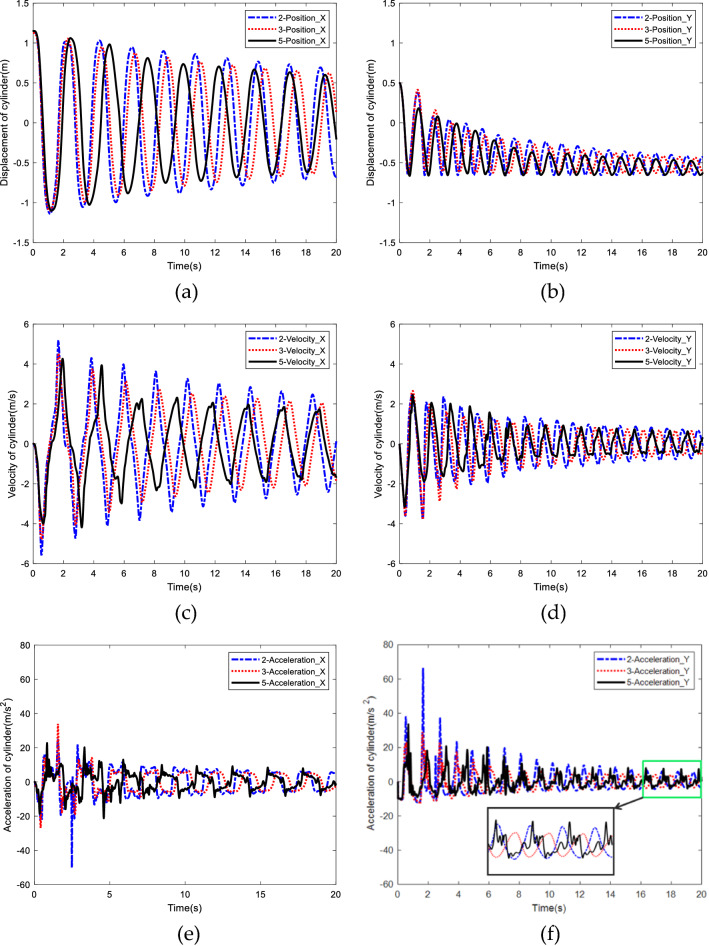
Comparison of different pattern of rigid body distribution. (**a**) Displacement in the X direction. (**b**) Displacement in the Y direction. (**c**) Velocity in the X direction. (**d**) Velocity in the Y direction. (**e**) Acceleration in the X direction. (**f**) Acceleration in the Y direction.

Figure 12 shows the comparison of displacement, velocity and acceleration of the rigid cylinder located at the end of cable under three different conditions.

As can be seen from the comparison of displacement, in Fig. 12a,b, when the total mass of the model remains unchanged, with the increase of the number of rigid bodies on the flexible cable, the displacement of the end cylinder decays faster with smaller amplitude. With respect to the comparison of velocity, the distribution of rigid bodies has great influence on the amplitude and period of velocity as shown in Fig. 12c,d, with the increase of the number of rigid bodies, the velocity cycle of the cylinder becomes larger. The difference is more obvious with longer simulation time. For example, the fifth peak of velocity is 10.2 s, 10.5 s and 11.9 s respectively with respect to two rigid-body, three rigid-body, and five rigid-body model. This phenomenon also infers that more rigid bodies may contribute to more stable swing speed of the cable system. From Fig. 12e,f, it can be seen from the first two cycles, the acceleration peak of the two-rigid body model is much greater than the other two cases, which is easy to cause the twining of the cable. With the increase of the number of rigid bodies, this phenomenon is obviously weakened. The attenuation of the acceleration of the five rigid-body model slows down with the increase of simulation time, and presents a strong nonlinear phenomenon.

### Influence of length of flexible element

The preliminary analysis implies that excessive number of segments of the flexible cable causes nonlinear phenomenon. It is speculated that the influence of flexible cable segments is similar to the number of flexible elements. Therefore, the effect of element length on the nonlinear characteristics of flexible cable motion is observed with the total length of the cable unchanged.

Three types of element length are set as shown in Table 4. Other parameters including material property and simulation conditions are consistent with the analysis above.

**Table 4:** Element length.

Element	Element_1	Element_2	Element_3
Element length	0.05 m	0.025 m	0.0125 m

**Figure 13:**
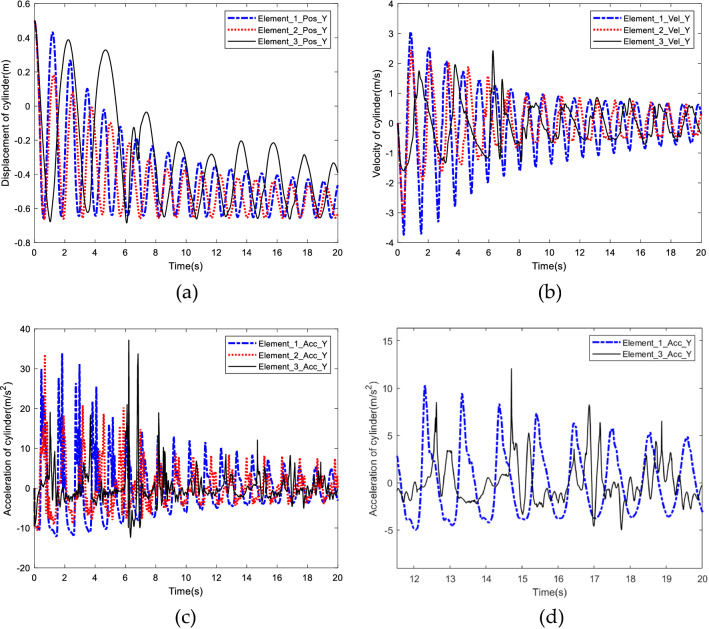
Comparison of different element length. (**a**) Comparison of displacement. (**b**) Comparison of velocity. (**c**) Comparison of acceleration. (**d**) Enlarged view of acceleration comparison between Element_1 and Element_3.

Comparison of displacement, velocity and acceleration of the end cylinder in the Y direction with different element length are shown in Fig. 13.

As shown in Fig. 13, with the decrease of element length, the nonlinear phenomenon becomes more obvious. The displacement with the smallest element length (Element_3) presents the largest swing amplitude, and velocity is no longer smooth but with sharp points and mutation phenomenon. The nonlinear phenomenon of acceleration is more obvious, and a large number of burrs appear in the curve. The simulation results imply that the shorter the element length, the stronger the nonlinear phenomenon of the rigid-flexible coupling system, and acceleration is the most sensitive to element length. In the rigid flexible coupling system, the addition of rigid bodies increases the complexity of flexible body motion. When there are fewer elements, the solution of the system is more stable and tends to converge.

**Figure 14:**
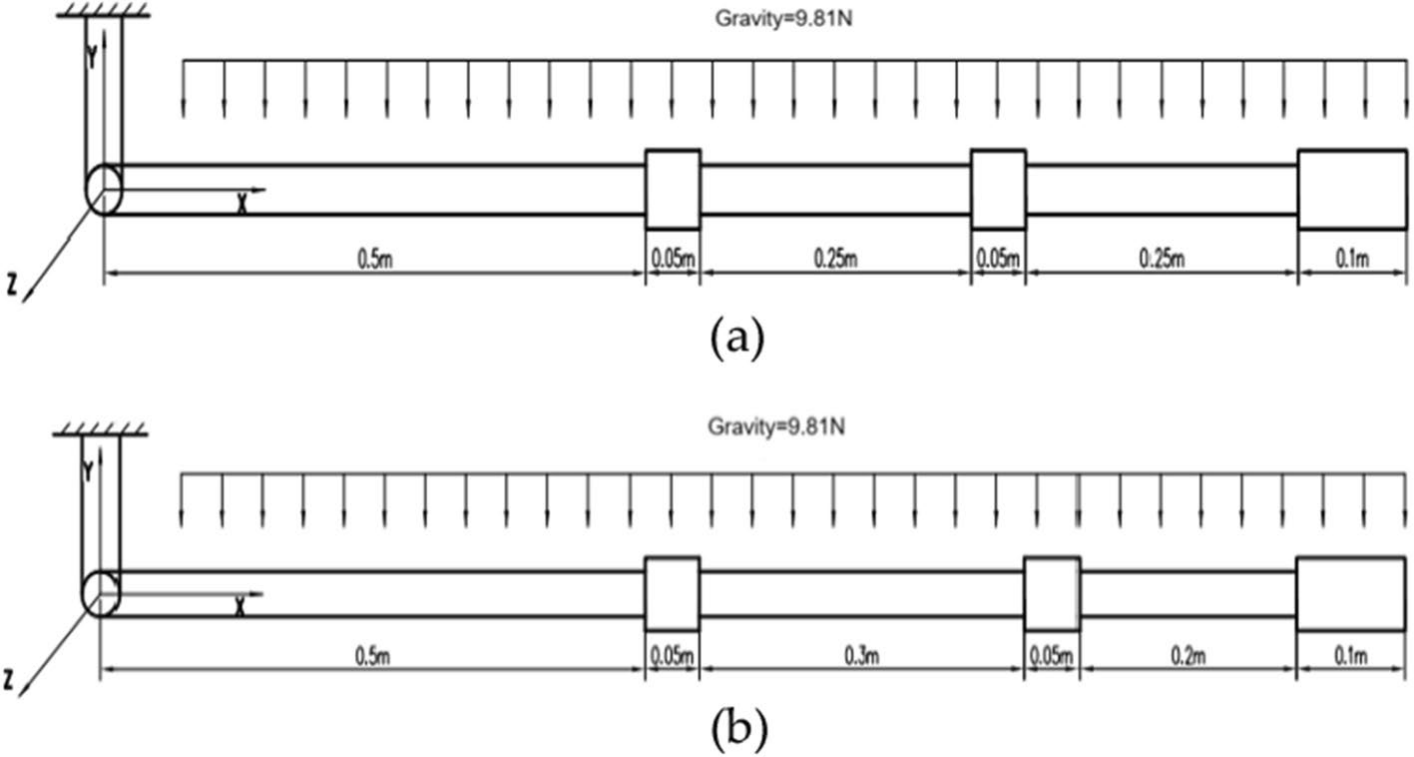
Schematic diagram of non-equidistant distribution of rigid body. (**a**) Equidistant distribution. (**b**) Non-equidistant distribution.

The above analysis is carried out under the condition of equidistant arrangement of rigid bodies. In order to explore the situation of non-equidistant distribution of rigid bodies, we select the three rigid-body model to design comparative experiments between equidistant and non-equidistant situations, the schematic diagram and parameters of model is shown in Fig. 14.

**Figure 15:**
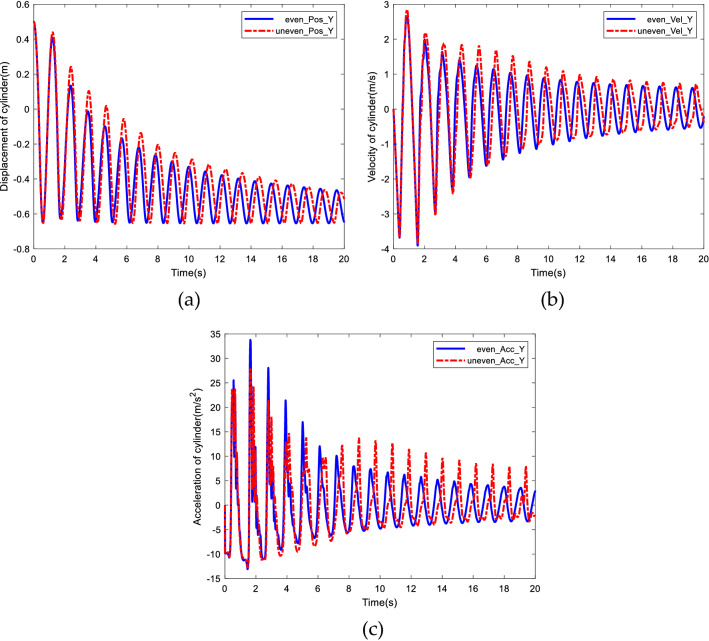
Comparison of equidistant and non-equidistant distribution of rigid bodies. (**a**) Comparison of displacement. (**b**) Comparison of velocity. (**c**) Comparison of acceleration.

The free swing process is simulated under the conditions of equidistant and non-equidistant arrangement of three rigid-body. Figure 15 shows the comparison of displacement, velocity and acceleration of the end cylinder in the Y direction.

Figure 15 shows that the swing amplitude, velocity and acceleration of the end cylinder with equidistant arrangement of rigid bodies is smaller compared with non-equidistant arrangement of rigid bodies, which implies that under the same conditions, the distribution of rigid bodies would affect the motion of the end of the cable, and the equidistant arrangement of rigid bodies will make the motion of the end of cable more stable (“Supplementary Information”).

## Conclusions

The rigid-flexible coupling cable model is established based on ANCF. The influence of number and distribution of rigid bodies, distribution patterns of rigid bodies and element lengths on the swing process is analyzed. When the total mass of rigid bodies remains unchanged, with more rigid bodies distributed, the cable system would have smaller swinging amplitude and be more stable. Equidistant distribution of the rigid bodies would weaken the swing amplitude and speed, and increase the stability of the system. The length of the flexible element directly affects the simulation accuracy of the motion of cable system. With shorter elements, the motion of cable system is more complex and nonlinear characteristics become more obvious. Meanwhile, this paper studies the discrepancies of different methods for solving large matrices of dynamic equations, and verifies the stability and practicability of MINRES method. In summary, with respect to rigid-flexible coupling cable system of large deformation and displacement, ANCF can be used to construct flexible elements efficiently, and this study is contributive to the influence of different factors on the movement of large deformation flexible cable body.

## Supplementary Information

Supplementary Information.

## Data Availability

The data used to support the findings of this study are available from the corresponding author upon request.
