# Supplementary material for: Dynamic modeling and simulation of rigid-flexible coupling cable system by absolute nodal coordinate formulation
Source: Sci Rep. 2022 Aug 9;12:13558. doi: 10.1038/s41598-022-17731-w (PMC9363493; doi:10.1038/s41598-022-17731-w)
Supplement: Supplementary file 1 — Supplementary Information. [file 41598_2022_17731_MOESM1_ESM.docx]

Model validation

Taking the one-rigid body model in this paper as an example, the convergence of the system solution under the influence of rigid bodies is studied. The flexible body is divided into 10, 20, 30, 40 and 50 elements and solved. Parameters are shown in Table S1, and the model diagram is shown in Figure S1.

**Table S1.** Parameters of the model

| **Material** | **Parameter** |
| --- | --- |
| cable length | L1=0.5m |
| Element diameter | D=0.01m |
| Element length | L=0.025m |
| Cross sectional area | A=7.85×10^-5^m^2^ |
| Section moment of inertia | I=4.91×10^-10^m^4^ |
| Elastic modulus | E=1.0×10^7^Pa |
| Element density | ρ=1.0×10^3^kg/m^3^ |
| Rigid-body height | H=0.1m |


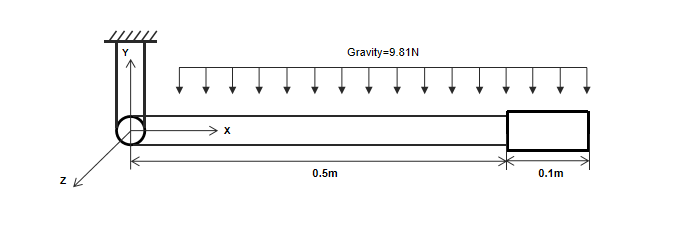


**Figure S1.** Rigid-flexible coupling model diagram with one rigid body at the end

(a) (b)

**Figure S2.** Comparison of different element length. (a) Rigid body velocity with different number of elements. (b) Rigid body acceleration with different number of elements.

It can be seen from Figure S2 that when the number of elements is more, the solution result is more divergent, and when the number of elements is less than 30, the solution of the system tends to converge. In the rigid flexible coupling system, the addition of rigid bodies increases the complexity of flexible body motion, and the more elements, the more difficult the solve of the system is and the more unstable the solution is. When the number of elements is lower than a certain value, the solution of the system will converge.
